# Effects of Resveratrol on Adipocytes: Evidence from In Vitro and In Vivo Studies

**DOI:** 10.3390/molecules29225359

**Published:** 2024-11-14

**Authors:** Matthew Terzo, Michael Iantomasi, Evangelia Tsiani

**Affiliations:** 1 Department of Health Sciences, Brock University, St. Catharines, ON L2S 3A1, Canada; 2 Centre for Bone and Muscle Health, Brock University, St. Catharines, ON L2S 3A1, Canada

**Keywords:** obesity, adipocytes, adipose tissue, resveratrol

## Abstract

Obesity, a prevalent global health issue, arises from an imbalance between caloric intake and energy expenditure, leading to the expansion of adipose tissue and metabolic dysfunction. White adipose tissue (WAT) stores energy as lipids, while brown adipose tissue (BAT) plays a pivotal role in energy dissipation through adaptive thermogenesis. Recent research initiatives have focused on finding strategies to decrease adipogenesis and fat mass accumulation and increase thermogenesis. Finding chemicals with anti-obesity properties would be beneficial. Resveratrol, a polyphenolic compound abundantly found in the skin of grapes and red wine, possesses anti-oxidant, anti-inflammatory, anti-cancer, and anti-obesity properties. This literature review examines the effects of resveratrol on adipocytes in culture and adipose tissue in animal models of obesity. The existing evidence indicates that resveratrol may exert its anti-obesity effects by inhibiting adipogenesis, promoting the apoptosis of mature adipocytes, reducing lipid accumulation, and increasing thermogenesis. Further research utilizing animal and clinical studies is required to understand in detail the anti-obesity potential of resveratrol.

## 1. Introduction

Obesity is defined as having a body mass index (BMI) of 30 kg/m^2^ or greater [1] and is a prominent hallmark of metabolic dysfunction. Obesity results from the accumulation of lipids and expansion of adipose tissue due to imbalanced energy intake and expenditure [2,3,4]. According to a World Health Organization (WHO) report, one in eight people in the world were living with obesity in 2022, representing a prevalent increase of more than twofold in adults since 1990. The increasing global prevalence of obesity and its related comorbidities, including cardiovascular disease, insulin resistance, and type 2 diabetes mellitus (T2DM), which collectively manifest as metabolic syndrome, has drawn attention to the importance and urgent need for developing anti-obesogenic strategies [1,5].

In mammals, white adipose tissue (WAT) and brown adipose tissue (BAT) make up the predominant forms of adipose tissue [6,7]. The primary function of WAT is to regulate lipid metabolism by storing energy as triglycerides. Lipid accumulation in adipocytes is accomplished by lipogenic genes, including fatty acid synthase (FASN) and acetyl-CoA carboxylase (ACC); these enzymes are regulated by transcriptions factors crucial for adipogenesis including proliferator-activated receptor gamma (PPARγ) and CCAAT/enhancer-binding protein alpha (C/EBPα) [8,9]. In the presence of caloric excess, individuals become obese by over-activation of adipogenesis and fatty acid synthesis [6,7]. In the presence of caloric deficit, lipolysis is stimulated, resulting in the production of fatty acids and glycerol molecules from stored triglycerides. Adipocytes also play an important endocrine role, secreting various adipokines, which play a role in regulating appetite, energy expenditure, and insulin sensitivity [10]. In obesity, adipocyte dysfunction leads to altered adipokine secretion and increased inflammation, contributing to overall metabolic dysfunction.

The function of BAT differs from WAT and is involved in adaptive thermogenesis, the production of heat through metabolic substrate utilization [11]. While brown adipocytes are more thermogenically active, the transdifferentiation or browning of white adipocytes is a physiological process that involves the adoption of a brown-like or beige phenotype. This process is stimulated by cold exposure, exercise, and certain anti-diabetic medications like metformin [12,13].

**Figure 1:**
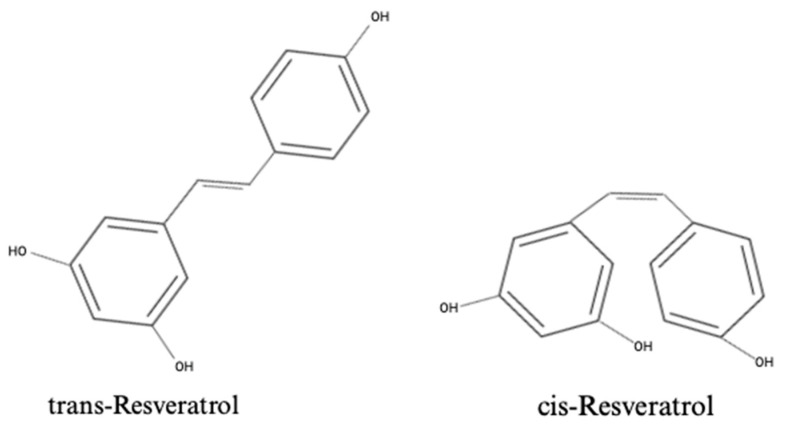
Chemical structures of the isoforms of resveratrol: trans-Resveratrol and cis-Resveratrol.

Resveratrol (RSV), 3,4′,5-trihydroxystilbene, is a naturally occurring polyphenolic compound commonly found in plants, including Vitis vinifera (grape vines), Arachis hypogaea (peanut plants), and Vaccinium species (blueberries, cranberries, and bilberries) [14,15]. RSV has a chemical formula of C_14_H_12_O_3_, and its structure is depicted in Figure 1. RSV exists as a stilbene compound, consisting of two phenolic rings bound together by a double styrene bond, forming a 3,5,4′-Trihydroxystilbene [16]. This molecular configuration gives rise to its two isomers, trans-3,5,4′-trihydroxystilbene (trans-RSV), being the biologically active form, and cis-3,5,4′-trihydroxystilbene (cis-RSV) [16]. RSV has been implicated in a wide range of biological functions [17,18], including anti-oxidant [19], anti-inflammatory [20], anti-cancer [21,22], and anti-diabetic functions [19,23], as well as protective effects against kidney disease [24]. RSV has also been suggested as a potential anti-obesity compound, mimicking the effects of energy restriction and leading to reduced body fat and improved insulin sensitivity [25].

We recognize there are a number of reviews on the effects of RSV on fat mobilization [26] and its anti-obesity properties [25,27]. In the current review, we summarized the effects of RSV on adipocytes by examining all available in vitro and in vivo animal and human studies from 2006 to the present (2024). We reported the effects of RSV on adipocyte signaling with emphasis on insulin signaling, not reported previously, which gives this review its novelty and offers new perspectives. We performed a PubMed search using the keywords “resveratrol and adipocytes”, “resveratrol and adipose tissue”, “resveratrol and brown adipocytes”, “resveratrol and beige adipocytes”, and “resveratrol and functional brown adipose tissue”. Articles were excluded if they were not specific to the topic or were not available in English. The articles are presented chronologically, and organized by species (murine studies presented first, followed by human studies).

## 2. The Effects of Resveratrol on Adipose Tissue

### 2.1. The Effects of Resveratrol on Adipocytes: In Vitro Evidence

The exposure of mature 3T3-L1 adipocytes to RSV (10, 20, and 40 µM) significantly decreased proliferation, attenuated lipid accumulation, and reduced triglyceride content [28]. These changes were associated with decreased C/EBPβ, CEBPα, FABP4, PPAR*γ*, Matrix Metalloproteinase-2, and 9 (MMP-2, MMP-9) protein levels (Table 1). Collectively, these findings suggest a potential decrease in adipogenesis by RSV.

Other researchers [29] exposed 3T3-L1 adipocytes to serum collected from monkeys fed a high-fat–sugar diet (HFSD) supplemented with or without RSV. Treatment with RSV-supplemented serum attenuated HFSD-induced inflammatory NFκB signaling, reduced IL-6 mRNA levels, increased Sirtuin 1 (SIRT-1) protein, and significantly increased insulin-induced cell surface GLUT-4 levels (Table 1).

In another study, the treatment of 3T3-L1 adipocytes with RSV reduced lipid accumulation with no significant effect on cytotoxicity [30]. RSV inhibited FASN by interacting with its ketoreductase (KR) domain (Table 1). Collectively, these findings indicate the potential of RSV to inhibit FASN activity and decrease adipocyte lipid storage.

RSV prevented hypoxia-inducible factor 1-alpha (HIF-1α) protein accumulation, and increased AMP-activated protein kinase (AMPK) and SIRT-1 protein levels in 3T3-L1 and primary adipocytes extracted from control mouse epididymal white adipose tissue (eWAT), both under environmental hypoxia (1% O_2_) and palmitic acid (PA)-induced hypoxia [31]. Collectively, these findings suggest the prevention of hypoxia in adipocytes by RSV, possibly mediated by SIRT-1.

Wang et al. [32] used stromal vascular cells (SVCs) isolated from the interscapular BAT of control C57BL/6 mice and found that RSV treatment significantly reduced lipid accumulation and decreased PPAR*γ* and aP2 protein levels (Table 1). RSV treatment also increased PRDM16, UCP1, PDH, and Cyto C protein content and increased adipocyte oxygen consumption, suggesting an increase in mitochondrial function and energy expenditure. Importantly, treatment with RSV increased the phosphorylation/activation of AMPK protein content, an effect that was abolished by dorsomorphin, a non-competitive inhibitor of AMPK, and AMPK-targeting siRNA. Moreover, the knockout of AMPK further eliminated RSV’s effects in increasing thermogenic biomarkers (PRDM16, UCP1, PDH, and Cyto C). Collectively, these findings suggest the potential of RSV to enhance thermogenic proteins, effects that may be mediated by AMPK. 

In a separate study utilizing 3T3-L1 adipocytes, RSV was reported to have cytotoxic effects above 10 µM, while at lower concentrations (1 to 10 µM) it reduced lipid accumulation and decreased the differentiation capacity of premature adipocytes [33]. In mature adipocytes, low-concentration of RSV also reduced PPAR*γ* and perilipin protein levels and attenuated isoproterenol- and TNF-α-induced lipolysis. Collectively, these findings demonstrate the ability of RSV to regulate adipogenesis, lipid metabolism, and adipocyte maturation.

Treatment of 3T3-L1 and human SBGS adipocytes with RSV protected against insulin-induced lipid accumulation and lipid droplet formation [34]. Additionally, RSV decreased citrate synthase (CS) activity, a measure of mitochondrial mass in primary and 3T3-L1 adipocytes. RSV also decreased ATPase family AAA domain-containing 3 (ATAD3), voltage-dependent anion channels (VDAC), ATP synthase F1 subunit alpha (ATP5a), ubiquinol-cytochrome c reductase core protein (UQCR), succinate dehydrogenase iron-sulfur subunit B (SDHB), and mitochondrially encoded cytochrome c oxidase I (MTCO1) protein levels. RSV increased phosphorylated ACC without affecting phosphorylated AMPK levels in both 3T3-L1 and primary adipocytes (Table 1). RSV decreased phosphorylated/activated Akt and its downstream effector, eukaryotic initiation factor 4E binding protein 1 (4E-BP1). Collectively, these findings demonstrate that RSV may decrease adipocyte mitochondrial biogenesis, in contrast to other studies. 

Nøhr et al. [35] conducted SILAC proteomic analysis on 3T3-L1 adipocytes, analyzing a total of 927 proteins post-treatment with RSV and LPS. LPS upregulated proteins involved in metabolite precursor generation, energy production, and the electron transport chain, such as NADH dehydrogenase and cyto c oxidase subunits. LPS downregulated proteins related to lipid metabolism, such as diglycerol acyltransferase 1 (DGAT1), stearoyl-CoA desaturase-1 (SCD1), emopamil binding protein (EBP), and protein glycosylation. RSV attenuated these LPS-induced effects by partially restoring levels of glycoproteins, SCD1, and DGAT1, while mitigating the LPS-induced reduction in adiponectin (Table 1). Furthermore, RSV counteracted the LPS-induced increase in pro-inflammatory markers, including STAT1, IFIT1, and other inflammatory response proteins such as S100-A11 and annexin A1. These findings demonstrate RSV’s potential to ameliorate the LPS-induced dysregulation of metabolic and pro-inflammatory proteins associated with obesity-related low-grade inflammation.

In another study, RSV significantly reduced lipid accumulation and increased SIRT-1, PPARγ, CPT1a, and PGC-1α mRNA and protein levels in 3T3-L1 adipocytes [36]. The use of SIRT-1 small interfering RNA (siRNA) abolished these increases and both SIRT-1 and PGC-1α siRNA led to significant triglyceride accumulation in adipocytes.

The exposure of 3T3-L1 adipocytes to RSV attenuated the PA-induced elevation of ATF6, PERK, IRE1α, GRP78, and CHOP mRNA, and IRE1, CHOP, and GRP78 protein levels [37]. RSV attenuated the PA-induced increase in leptin, resistin, TNF-α, IL-1β, SREBP1c, and PPAR*γ* mRNA levels, while it increased adiponectin, PPARα, and SIRT-1 mRNA levels. Collectively, these findings suggest the potential of RSV to reduce inflammation and ER stress induced by the exposure of adipocytes to fatty acids.

Isolated SVCs from the inguinal white adipose tissue (iWAT) and BAT from NMRI mice, exposed to RSV through maternal supplementation during the weaning period, were analyzed [38]. RSV treatment did not significantly increase thermogenic gene expression (UCP1, CPT1B, PPARGC1α, PPARGC1B, PPARA, PRDM16) in iWAT-derived adipocytes. Importantly, only PPARGC1α mRNA levels significantly increased in male brown adipocytes, while all other examined genes (UCP1, CPT1B, PPARGC1B, PPARA, PRDM16) showed no significant change (Table 1). Collectively, these findings suggest that early maternal RSV supplementation does not significantly alter thermogenic gene expression in offspring primary adipocytes, possibly due to an insufficient dose. Further research is needed to explore this question.

Primary brown adipocytes isolated from control mice demonstrated increased UCP1, PGC-1α, and SIRT-1 mRNA levels when treated with RSV (Table 1) [39]. Primary white adipocytes, isolated from subcutaneous mouse adipose tissue, had increased UCP1, FNDC5, and SIRT-1 mRNA levels when treated with RSV. In primary adipocytes, isolated from the visceral adipose tissue of mice, RSV treatment increased SIRT-1 mRNA levels. Importantly, sirtinol, a SIRT-1 inhibitor, abolished RSV’s effects in all primary adipocyte cultures, indicating a SIRT-1-dependent mechanism.

3T3-L1 adipocytes treated with RSV resulted in a significant reduction in triglyceride content and increased FFA and glycerol content, suggesting that RSV may promote lipolysis [40]. Additionally, RSV decreased 2′,7′-Dichlorodihydrofluorescein (DCF) fluorescent intensity, indicating a reduction in ROS. Collectively, these findings suggest the potential of RSV to modulate lipid metabolism and storage in adipocytes, as well as to act as an anti-oxidant compound. 

A dose-dependent reduction in lipid accumulation, coupled with an increase in UCP1, PPAR*γ*, and PGC-1α protein levels, was seen in 3T3-L1 adipocytes treated with RSV [41]. Importantly, the use of the mammalian target of rapamycin (mTOR) inhibitors (MHY1485 and rapamycin) attenuated the increased protein levels of UCP1, PPAR*γ*, and PGC-1α in adipocytes (Table 1). Collectively, these findings demonstrate the potential of RSV to induce brown-like phenotypes in white adipocytes and elevate mitochondrial biogenesis, mediated by mTOR activation.

Exposure of 3T3-L1 adipocytes to RSV and resveratrol butyrate esters (RBEs) resulted in a decrease in proliferation and lipid accumulation (Table 1) [42]. Importantly, exposure to RSV and RBEs reduced PPAR*γ*, C/EBP, FABP4, and FASN mRNA levels. Additionally, the treatment of adipocytes with RSV significantly increased the phosphorylation/activation of AMPK.

The effects of RSV and its metabolites trans-resveratrol-3-O-glucuronide (R3G), trans-resveratrol-4′-O-glucuronide (R4G), and trans-resveratrol-3-O-sulfate (R3S) were examined and all were found to reduce lipid accumulation in premature and mature 3T3-L1 adipocytes [43]. RSV R3G, R4G, and R3S reduced C/EBPβ mRNA levels in premature adipocytes (Table 1), and only R3S decreased C/EBPα, PPAR*γ*, and LPL mRNA levels. In mature adipocytes, RSV increased ATGL, CPT1b, SIRT-1, and PGC1α mRNA (Table 1), while R4G increased HSL, and R3G reduced FASN mRNA in mature adipocytes. Collectively, these results indicate that resveratrol and its metabolites may play a role in regulating adipogenesis, lipogenesis, and mitochondrial biogenesis.

Eseberri et al. [44] treated 3T3-L1 adipocytes with RSV and RSV metabolites (R3G, R4G, and R4S) to investigate their effect of adipokine regulation [44]. RSV reduced leptin mRNA and secretion levels, while reducing adiponectin mRNA levels. Importantly, R3G and R4G increased apelin and visfatin mRNA levels. R3S reduced leptin mRNA, while it increased apelin and visfatin mRNA levels in adipocytes. Collectively, these findings suggest that RSV and its metabolites may regulate adipokines, important for fat metabolism, emphasizing the need for further examination in animal and human studies.

In another study the same group (Eseberri et al. [45]) treated 3T3-L1 adipocytes with RSV and RSV metabolites and found that RSV, R3G, and R4G increased miR-155 levels, suggesting that miR-155 is involved in the action of these compounds. Additionally, RSV and R4G significantly reduced cAMP response element-binding Protein 1 (CREB1) and Kruppel-Like Factor 5 (KLF5) mRNA levels, two important genes involved in adipogenesis. RSV reduced CEBPβ mRNA and protein levels, while transfection with miR-155 inhibitor attenuated the reduction in CEBPβ. R3S significantly decreased sterol regulatory element binding transcription factor 1 (SREBF1) and Retinoid X Receptor Alpha (IXRα) mRNA activity. Additionally, amongst many various microRNA (miR-155, miR-27b, miR-27a, miR-130b, miR-31, miR-326, miR-144, miR-205, and miR-244), none were affected by R3S. These findings suggest that RSV and its metabolites can regulate adipogenesis. While RSV and its glucuronide metabolites (R3G and R4G) act through miR-155, the sulfate metabolite (RS3) exerts its anti-adipogenic effects through a different, miR-155-independent mechanism.

The treatment of premature 3T3-L1 adipocytes with RSV and DR2 resulted in decreased lipid accumulation, PPAR*γ*, FASN, C/EBPα, and p38 protein levels, while phosphorylated AMPK levels were increased in premature adipocytes [46]. Taken together, these results indicate that RSV and DR2 may regulate lipogenesis and adipogenesis, mediated by AMPK.

Ku et al. [47] examined the effects of RSV on primary brown preadipocytes isolated from the interscapular brown adipose tissue (iBAT) of 4-week-old chow-fed Sprague Dawley rats. The treatment of adipocytes with this RSV increased UCP1, p-AMPK, and estrogen receptor-α (ER-α) protein contents.

Researchers used SVCs isolated from the abdominal WAT of non-diabetic female humans [48]. RSV attenuated the conjugated linoleic acid (CLA)-induced phosphorylation/activation of extracellular signal-regulated kinase (p-ERK), c-Jun N-terminal kinase (p-JNK), phospholipase A2 (p-PLA2), and activating transcription factor 3 (ATF-3), all associated with cellular stress (Table 1). RSV also reduced CLA-induced inflammation, shown by lower levels of interleukin-6 (IL-6), IL-8, IL-1β, cyclooxygenase-2 (COX-2) mRNA, and reduced prostaglandin F2α (PGF2α) secretion. Additionally, RSV decreased reactive oxygen species (ROS) production and Cytokine Signaling 3 (SOCS-3) mRNA levels in CLA-stimulated adipocytes. Importantly, RSV mitigated CLA-mediated glucose uptake reduction, increased adiponectin and SIRT-1 levels, and attenuated the CLA-induced decrease in peroxisome proliferator-activated receptor gamma (PPARγ), while reducing lipid accumulation and free fatty acid (FFA) uptake. These findings suggest RSV’s potential to attenuate CLA-induced insulin resistance and modulate adipocyte lipid content by mitigating inflammation and stress.

Another study by Fischer-Posovszky et al. [49] used human Simpson–Golabi–Behmel syndrome (SGBS) preadipocytes and found that RSV decreased differentiation and lipid accumulation. In premature adipocytes, RSV treatment inhibited cell survival and thymidine incorporation, indicating a reduction in proliferation. The treatment of mature adipocytes with RSV increased basal and insulin-stimulated glucose uptake and decreased insulin-stimulated glucose incorporation into intracellular lipids. Importantly, RSV downregulated PPAR*γ*, GLUT-4, FASN, and Acetyl-CoA Carboxylase (ACC) mRNA levels in mature adipocytes. Furthermore, RSV treatment decreased IL-6 and IL-8 mRNA and protein levels. Importantly, the effects of RSV were determined to be SIRT-1-dependent, as knockdown models of SIRT-1 abolished the RSV-induced effects.

The treatment of human primary adipocytes (SGBS) with RSV reduced plasminogen activator inhibitor-1 (PAI-1) mRNA and protein levels, both in basal and macrophage-conditioned media (MCM)-stimulated conditions [50]. This anti-inflammatory effect of RSV was partially SIRT-1-independent, as sirtinol, a SIRT-1 inhibitor, did not prevent the reduction in PAI-1 levels (Table 1). Furthermore, RSV’s action was independent of AMPK, as its knockout did not block its anti-inflammatory effects. Similarly, phosphoinositide 3-kinase (PI3K) was ruled out as a regulator, as RSV still reduced MCM-induced PAI-1 levels even in the presence of the PI3K inhibitor LY294002 [50].

Another group of researchers [51] utilized SGBS human primary adipocytes and human Tamm–Horsfall Protein THP-1 macrophages and found that the exposure of adipocytes to MCM or direct co-culture elevated IL-6, IL-8, and MCP-1 mRNA and secretion levels (Table 1). RSV treatment significantly reduced these pro-inflammatory markers in both MCM-exposed and co-cultured adipocytes. Pretreatment with LY294002, a PI3K inhibitor, blocked RSV’s reduction in MCP-1, but not IL-6 and IL-8, suggesting that MCP-1 suppression may involve PI3K signaling. Additionally, RSV’s inhibition of IL-6, IL-8, and MCP-1 was comparable to that of SC-514, a selective NFκB inhibitor, indicating that RSV acts similarly to an NFκB inhibitor, demonstrating strong anti-inflammatory effects in human adipocytes.

Tran et al. [52] used human placenta, adipose tissue, and skeletal muscle stimulated with pro-inflammatory cytokines TNF-α and IL-1β, LPS, and poly(I:C) to induce a model of gestational diabetes (GD). Treatment with RSV significantly reduced pro-inflammatory secretion in placenta and omental adipose tissue. Additionally, RSV increased the phosphorylation of insulin receptor substrate-1 (IRS-1), increased GLUT4 protein content, and increased glucose uptake in human skeletal muscle stimulated with TNF-α, LPS, or poly(I:C) (Table 1). Collectively, these findings suggest the potential of RSV to attenuate inflammation and insulin resistance in muscle cells.

**Figure 2:**
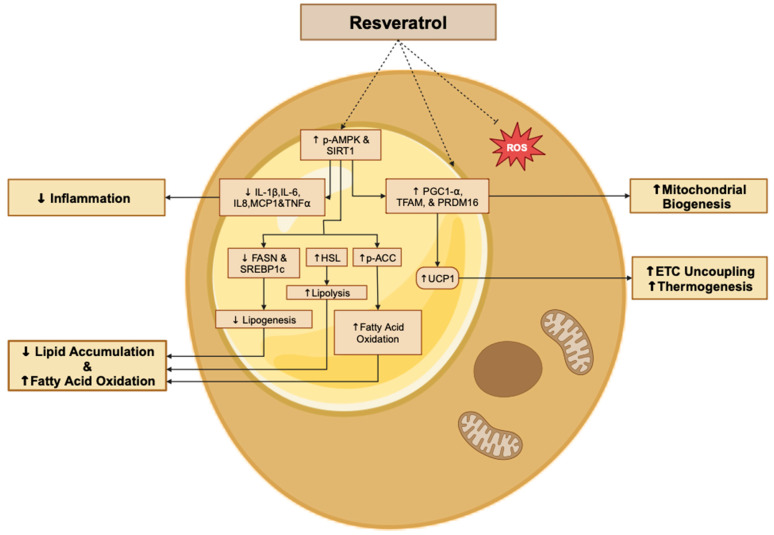
Summary of the effects of resveratrol in adipocytes. The figure, created using www.BioRender.com, is based on the data from the studies [28,29,30,31,35,37,40,49]. ↑: increased, ↓: reduced, and p: phosphorylated.

Overall, the in vitro evidence summarized in the above section indicates the strong potential of RSV treatment to decrease lipid accumulation, reduce lipogenesis, attenuate insulin resistance, decrease ROS, induce browning, and attenuate pro-inflammatory responses in both stable adipocyte cultures and primary adipocytes (Figure 2).

### 2.2. The Effects of Resveratrol on Adipose Tissue: In Vivo Evidence

A 2011 study found that ad libitum RSV administration protected mice against HFD-induced weight gain and fat mass increase in epididymal, perirenal, mesenteric, and retroperitoneal adipose tissue [53]. RSV reduced the levels of triglycerides, glucose, TNFα, and MCP1 in the plasma of HFD mice. RSV protected against HFD-induced increases in GalR1/2, PKCδ, Cyclin-D, E2F1, p-ERK, and adipogenic markers PPARγ2, C/EBPα, SREBP-1c, FASN, LPL, aP2, and leptin in the eWAT of mice, indicating a potential reduction in adipogenesis and fatty acid synthesis. Importantly, RSV attenuated HFD-induced increases in pro-inflammatory cytokines TNFα, IFNα, IFNβ, and IL-6, as well as their upstream signaling molecules, TLR2/4, MyD88, Tirap, TRIF, TRAF6, IRF5, p-IRF3, and NF-kB in eWAT. Collectively, these findings suggest the anti-inflammatory and anti-adipogenesis potential of RSV in mice.

A separate study found that RSV supplementation significantly decreased retroperitoneal and eWAT, despite no changes in food intake, lean mass, or total body weight [54]. The serum lipid profile and plasma glucose levels were improved in RSV-treated mice. Importantly, RSV increased UCP1, SIRT-1, phosphatase, and tensin homolog (PTEN), and bone morphogenetic protein 7 (BMP-7) mRNA levels in BAT, and an increase in oxygen consumption was reported. These findings suggest that RSV reduces adipose tissue mass while promoting the expression of thermogenic genes.

The hypolipidemic effects of RSV supplementation in apoE-deficient mice was examined [55]. RSV reduced body weight gain and reduced plasma triglyceride, total cholesterol, LDL-C, apolipoprotein B (apoB), and the apoB/apoA-I ratio while concomitantly increasing plasma HDL-C (Table 2).

CD1 female HFD mice supplemented with RSV had reduced weight gain and iWAT index (iWAT mg/g body weight), despite no significant difference in food consumption [32]. Importantly, RSV induced a brown-like phenotype, and increased p-AMPK, UCP1, PRDM16, and Cyto C mRNA and protein levels in iWAT, as well as oxygen consumption levels (Table 2). The respiratory exchange ratio (RER)(VCO_2_/VO_2_) was decreased in RSV-supplemented mice, indicating increased use of fatty acids (Table 2). Collectively, this evidence shows that RSV may induce browning within WAT and enhance overall energy expenditure.

RSV attenuated HFD-induced total body weight gain and the specific accumulation of epididymal and subcutaneous adipose tissue associated with obesity [33]. Importantly, RSV significantly reduced hepatic mass, while the weights of all other examined tissues (heart, muscle, and kidneys) were not affected (Table 2). Adipocyte size was reduced, and the number of adipocytes increased in subcutaneous and epididymal adipose tissue extracted from RSV-treated mice. Collectively, these findings suggest the strong potential of RSV to attenuate the consequences associated with HFD consumption and protect against obesity.

RSV attenuated HFD-induced hypoxia, and decreased HIF-1α, inositol-requiring protein-1α (IRE1α) and eukaryotic translational initiation factor 2α (eIF2α) (two indicators of ER stress) mRNA and protein levels in mouse eWAT [31]. Furthermore, RSV attenuated collagen 3α (Col3α), collagen 6α (Col6α), elastin, and lysyl oxidase (LOX) mRNA and reduced the HFD-induced inflammatory response (TNF-α, IL-6, MCP-1 and F4/80), indicating reduced collagen deposition and anti-inflammatory properties.

Researchers [56] found that RSV supplementation in CD1 mice attenuated HFD-induced weight gain, despite no difference in caloric intake. RSV increased the number of adipocytes in iBAT and increased UCP1, PRDM16, Cyto C, and phosphorylated AMPK protein levels. These findings suggest the potential of RSV to enhance the thermogenesis of BAT, which may contribute to metabolic inefficiency, ultimately protecting against HFD in mice.

The exposure of pregnant HFD mice to RSV attenuated total body weight and WAT mass increase, while improving serum triglyceride and insulin levels [57]. In the offspring of HFD-RSV mice, total body mass and WAT gain were attenuated. At weaning, adipocyte size was reduced, and UCP1, PRDM16, Elov13, PGC1-α, and CD137 mRNA, as well as UCP1, PRDM16, Cyto C, SIRT-1, and p-AMPK protein, were increased in the iWAT of RSV-supplemented pups (Table 2). Post-weaning, 14-week RSV supplementation protected against obesity in pups as HFD total body mass and WAT gain were attenuated, and insulin sensitivity, blood glucose, and lipid profile were improved. Importantly, RSV administration increased oxygen consumption and heat production. These findings suggest the potential of RSV to protect against obesity in adult and offspring mice.

RSV supplementation in C57BL/6 mice attenuated elevated fat mass and improved insulin sensitivity, glucose tolerance, and the serum lipid profile in HFD mice [58]. Moreover, RSV attenuated the HFD-induced pro-inflammatory response (MCP-1, TNF-α, and IL-6 serum protein) and reduced F4/80, CCR2, MCP-1, TNF-α, and IL-6 mRNA levels in SAT and VAT. Additionally, RSV increased IRS-1 and GLUT4 mRNA and protein, while increasing p-Akt protein levels in SAT and VAT (Table 2).

In another study, RSV attenuated total body weight and WAT gain, in addition to improving insulin sensitivity and glucose tolerance [59]. Moreover, RSV decreased adipocyte size, increased adipocyte number, and induced browning in the WAT of HFD mice. Importantly, RSV altered the fecal microbiota of HFD mice and reversed HFD-induced gut dysbiosis. RSV fecal microbiota transplantation (FMT) altered the HFD-recipient mice gut microbiome, with the changes being consistent with the control mice. Importantly, RSV-FMT protected against HFD weight and iWAT mass gain and improved glucose tolerance and insulin sensitivity, while SIRT-1 protein was increased (Table 2). These findings suggest the anti-obesity potential of RSV and its associated gut microbiome changes, which may be transferable to recipient mice.

Researchers [37] found that RSV reduced body mass, SAT, and VAT gain in high-fat–sugar diet (HFSD) mice. RSV improved glucose tolerance, insulin sensitivity, and serum lipid profile, while attenuating RSV ER stress gene mRNA levels (ATF6, PERK, IRE1α, GRP78, and CHOP), adipokines (leptin, resistin, and adiponectin), pro-inflammatory factors (TNF-α, MCP-1, IL-6 and IL-1β), and lipid metabolism markers (SIRT-1, SREBP1c, PPARα, and PPARγ) in SAT and VAT extracted from HFSD mice.

RSV administered intraperitoneally for two days in mice reduced the RER and increased AMPK and ACC1 mRNA levels, while CACT protein levels increased in adipose tissues [60]. These data suggest the potential of RSV to enhance fatty acid transport and oxidation through AMPK activation in mice.

Andrade et al., 2019 [39] found that RSV attenuated HFD-induced visceral and subcutaneous adipose tissue mass increase, increased plasma adiponectin, improved insulin and glucose sensitivity, and reduced total cholesterol. Additionally, RSV increased BAT mass and thermogenic mRNA (UCP1, PGC-1α, and PRDM16) and SIRT-1 mRNA in mouse BAT. Furthermore, FNDC5, UCP1, and PRDM16 mRNA were upregulated in mouse subcutaneous and visceral adipose tissues (Table 2).

According to another study [61], RSV improved glucose tolerance in diabetic (db/db) mice, increased Cidea, Ppara, Pparg, and PRDM16 mRNA in iWAT, and Cidea, PRDM16, Ppargc1a, and Dio2 mRNA in BAT, with UCP1 mRNA and protein levels elevated in both tissues. Lithocholic acid (LCA), a ligand of takeda G-protein coupled receptor 5 (TGR5), was higher in plasma and fecal samples of RSV-treated mice, suggesting an LCA-TGR5 interaction may mediate these effects. RSV altered the gut microbiome by preventing an increase in *Firmicutes* and the *Firmicutes*/*Bacteroidetes* ratio, commonly seen in obesity. Antibiotic use abolished these microbiome changes and partially blocked RSV’s effects on thermogenic genes and glucose tolerance. Additionally, FMT showed that the benefits of RSV were transferable and dependent on the gut microbiome.

The intraperitoneal injection of RSV in HFD C57BL/6J mice attenuated whole-body weight gain and iWAT mass gain [40]. Importantly, adipocyte size was reduced in iWAT extracted from RSV-supplemented HFD mice. RSV reduced FFA and glycerol content within iWAT.

RSV supplementation altered the C57BL/6J mice gut microbiome (increased *Bacteroidetes* and decreased *Firmicutes* phylum) [62]. Additionally, RSV FMT attenuated HFD-induced weight gain, reduced WAT, and increased BAT in recipient mice, while energy intake was unchanged. Importantly, RSV FMT improved glucose and insulin tolerance and reduced SREBP1c, FAS, SCD1, and ACACa mRNA levels, while increasing CPT 1-α, PDK4, MCAD, UCP1, PRDM16, PGC1-α, SIRT-1, and PPARα mRNA levels in WAT. Likewise, CPT-1α, PDK4, PPARα, UCP1, PRDM16, PGC1-α, and SIRT-1 were all increased by RSV FMT in BAT from HFD mice [62]. Taken together, these results suggest that RSV-mediated gut microbiome alteration may induce browning in WAT and enhance BAT thermogenic properties, protecting against obesity and T2DM.

Lam et al., 2023 [46] supplemented HFD C57BL/6J mice with dihydro-resveratrol (DR2), which significantly attenuated weight gain and percent-weight change. Higher dose DR2 supplementation (80 mg/kg) attenuated elevated blood glucose concentrations associated with HFD (Table 2). Importantly, DR2 attenuated elevated MCP1 mRNA expression levels and possessed increased the phosphorylation/activation of AMPK protein levels in iWAT extracted from HFD mice.

In a separate study, Macarulla et al. [63] administered RSV to Sprague Dawley rats and found that RSV attenuated HFSD-induced eWAT, perirenal adipose, mesenteric adipose, and subcutaneous adipose tissue weight gain. RSV administration did not affect BAT, gastrocnemius muscle, or liver weights. Additionally, the serum parameters analyzed, including cholesterol, triacylglycerols, free fatty acids, and glucose, were not affected by RSV treatment.

Alberdi et al., 2011 [64] supplemented Sprague Dawley rats with RSV and found that RSV reduced perirenal, epididymal, mesenteric, and subcutaneous WAT-specific mass gain (Table 2). Additionally, RSV reduced the activity of glucose-6-phosphate dehydrogenase (G6PDH), FASN, and ACC, and decreased hormone-sensitive lipase (HSL) mRNA levels (Table 2). Collectively, these findings demonstrate the potential of RSV to protect against obesogenic HFD by reducing fatty acid uptake and repressing lipogenesis.

RSV supplementation in male rats increased mitochondrial biogenesis gene (TFAM, COX2, PPARα/β, and PGC-1α) mRNA and increased SIRT-1 mRNA in iBAT [65]. Additionally, RSV-treatment in mice increased UCP1 protein levels in the iBAT of mice. These data indicate that RSV may upregulate thermogenic genes in the BAT of mice.

RSV protected against diet-induced body weight gain in male rats, while in female rats, it reduced diet-induced increases in omental fat mass, plasma and adipose insulin levels, and plasma triglyceride levels [66]. Importantly, RSV abrogated diet-induced elevations in adipose tissue levels of TNF-α, alanine transaminase (ALT) and aspartate aminotransferase (AST); however, this was only in female rats. On the contrary, RSV reduced diet-induced elevations in adipose levels of malondialdehyde (MDA) and cytokines, IL-6, IL-10, and IL-18 (Table 2). Additionally, RSV reversed diet-induced increases in PI3K and inducible nitric oxide synthase (iNOS) mRNA in the adipose tissue of males and females; elevations in the mRNA expression of Akt, PPAR*γ*, and endothelial nitric oxide synthase (eNOS) were only attenuated in females (Table 2). Additionally, Nrf2 expression, which was elevated following high fructose feeding, was reduced, but only in males.

The administration of RSV to male streptozotocin (STZ) and nicotinamide (NA)-induced diabetic Wistar rats attenuated insulin resistance and had hypoglycemic and anti-oxidant effects (Table 2) [67]. RSV reduced blood glucose and increased insulin sensitivity. Importantly, RSV increased plasma superoxide dismutase (SOD) activity and reduced forkhead-related transcription factor (FOXO1) and FOXO3 mRNA levels in iWAT tissue.Collectively, these findings suggest the potential of RSV to act as a hypoglycemic and anti-oxidant agent. 

Ardid-Ruiz et al. [68] supplemented obese rats with RSV and found reduced body weight and fat accumulation. Importantly, total levels of lipids were reduced in the liver, muscle, and eWAT. A significant reduction in serum leptin, glucose, insulin, and triglycerides was detected (Table 2), indicating improved leptin and insulin signaling. Importantly, SIRT-1 levels increased in the liver and muscle, but not eWAT in obese mice. These therapeutic effects of RSV were demonstrated to be dependent on SIRT-1 levels, as increases in SIRT protein levels were observed in isolated tissue samples. RSV supplementation in mice resulted in the detection of 10 different metabolites in serum, predominantly phase II conjugates, with glucuronides being more abundant than sulfates, while the parent compound was not detected.

RSV supplementation attenuated the HFSD-induced total weight, and WAT mass gain and also increased nephroblastoma overexpressed protein (NOV/CCN3) gene expression in eWAT compared to the obese non-supplemented group [69]. These findings suggest the potential of RSV to modulate adipokine NOV/CCN3 levels; however, its broader anti-diabetic implications require more study.

The administration of ligand-coated trans-RSV encapsulated nanoparticles (L-Rnano) to obese mice reduced body weight, total fat mass, and specific fat masses in gonadal WAT (gWAT) and (iWAT) [70]. Additionally, trans-RSV significantly reduced adipocyte size in iWAT and significantly increased UCP1 protein content (Table 2). RSV improved insulin sensitivity and lowered fasting plasma insulin and glucose concentrations, and decreased plasma leptin concentrations. RSV reduced total cholesterol and LDL cholesterol in obese mice. Collectively, these findings demonstrated the potential of RSV to protect against obesity via targeted drug delivery.

Jimenez-Gomez et al., 2013 [29] examined the effects of a 2-year supplementation with RSV in adult male rhesus monkeys and found that RSV treatment attenuated increases in LDL cholesterol levels. Interestingly, RSV treatment counteracted diet-induced alterations in inflammation-related pathways, stress responses, and metabolic processes, including electron transport chain-related genes in subcutaneous WAT. RSV increased the number of adipocytes and decreased adipocyte size in isolated visceral WAT (vWAT), but not in subcutaneous WAT (sWAT). Additionally, RSV increased SIRT-1 protein levels in vWAT but not in sWAT. Resveratrol reduced NF-kB-activated protein levels and its downstream target genes, IL-6 and IL-1β mRNA levels, in the visceral fat of the animals (Table 2). Additionally, RSV supplementation increased IRS-1, phosphorylated/active AKT, and Glut-4 protein levels in RSV- HFSD monkeys, demonstrating improved insulin signaling in vWAT.

**Figure 3:**
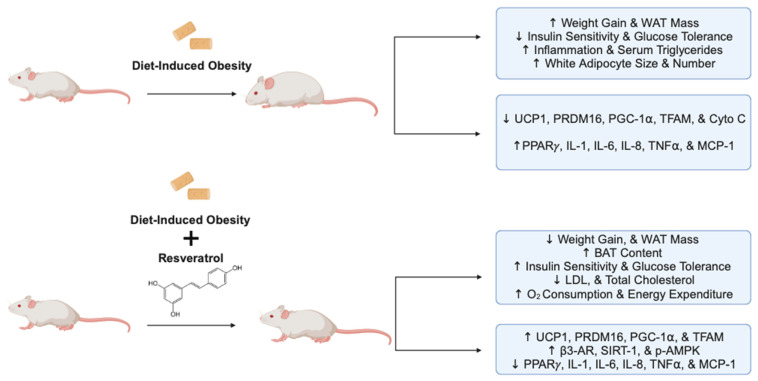
Summary of the effects of resveratrol in animal models of obesity and insulin resistance. The figure, created using www.BioRender.com, is based on the data of the in vivo studies [29,32,33,34,57,58,60,70,71]. ↑: increased, ↓: reduced, and p: phosphorylated.

Overall, the evidence above indicates the strong potential of RSV administration to exert anti-obesity effects, increase insulin sensitivity, and improve glucose tolerance in animal models of obesity and insulin resistance (Figure 3).

### 2.3. The Effects of Resveratrol on Adipose Tissue: Evidence from Human In Vivo Studies

In a randomized, double-blinded, placebo-controlled clinical study, Yoshino et al. orally administered RSV (75 mg/kg) in non-obese women with normal glucose tolerance for 12 weeks [72]. Supplementation with RSV had no effect on body composition, metabolic markers, inflammatory indicators, insulin signaling, energy metabolism, or cardiovascular function. Likewise, SIRT-1 NAMPT, PPARGC1A, and UCP3 mRNA levels were unaffected by RSV in muscle and adipose tissues (Table 3). Similarly, the protein content of phosphorylated/activated AMPK was not affected by RSV treatment in human gastrocnemius muscle biopsy samples. This study found that resveratrol supplementation in non-obese women had no significant metabolic effects.

Researchers, in another randomized and placebo-controlled cross-over clinical study [71], treated 13 male type 2 diabetic patients; first-degree relatives with RSV (150 mg/day) for 30 days and found that plasma concentrations of RSV were approximately 300 ng/mL (1.31 µM), and concentrations of dihydro-RSV were higher, around 600 ng/mL (2.61 µM) (Table 3). RSV supplementation had no effect on insulin sensitivity, as determined using a two-step hyperinsulinemic-euglycemic clamp. Additionally, RSV supplementation significantly increased skeletal muscle mitochondrial state three respiration on a lipid-derived substrate and with parallel electron input to both complex I and II (Table 3), indicating that RSV significantly enhances mitochondrial function. However, RSV did not affect ectopic fat accumulation, cardiac function, or brown adipose tissue activation in this study.

Obese males, with metabolic syndrome, orally received RSV (1 g, twice daily for 30 days) in a randomized placebo-controlled clinical trial [73]. RSV improved insulin sensitivity and glucose homeostasis exclusively in Caucasian participants (Cauc. pts.). Furthermore, in Caucasian subjects, RSV supplementation significantly increased the relative abundance of several bacterial taxa, most notably, *Akkermansia muciniphila*, *Barnesiella*, and *Odoribacter*. This effect was not observed in the placebo group or in non-Caucasian participants.

In obese humans, Andrade et al. [39] found that administration of trans-RSV upregulated SIRT-1 mRNA levels and thermogenic and mitochondrial genes UCP1, PGC-1α, and PRDM16 in human subcutaneous adipose tissue biopsy (Table 3). These findings suggest RSV’s potential to enhance thermogenic genes within the adipose tissues of obese individuals. 

A combination of RSV (250 mg) supplementation and lifestyle changes in a randomized controlled clinical trial involving 25 obese individuals significantly reduced participants’ body weight, BMI, waist circumference, fat mass, and both systolic and diastolic blood pressures [74]. This combined approach reduced serum LDL cholesterol and triglyceride levels and increased HDL cholesterol levels. Importantly, fasting blood glucose levels were decreased, and insulin sensitivity (HOMA-IR) was improved by RSV supplementation. Furthermore, a significant reduction in serum inflammatory markers, including TNF-α, CRP, and IL-6, and oxidative stress markers, such as MDA, SOD, and GPx activity, was observed in RSV-treated participants (Table 3). These findings suggest that RSV supplementation combined with lifestyle modifications can significantly mitigate the consequences associated with metabolic syndrome.

Overall, the human clinical studies reviewed above indicate the anti-obesity and anti-diabetic potential of RSV. Specifically, RSV may improve insulin sensitivity and reduce fasting plasma glucose levels in obese individuals. There is a need for more organized clinical studies investigating the effects of RSV in obese, insulin-resistant, and T2DM patients prior to its recommended use in the treatment of these conditions.

## 3. Discussion and Conclusions

The studies discussed in this review provide strong evidence that RSV modulates adipogenesis, lipid storage, and thermogenic function in adipocytes, while it may exert anti-obesity and anti-diabetic properties in animals and humans. 

Several studies indicated that RSV treatment reduced adipocyte maturation [33,43,46] and induced apoptosis in premature white adipocytes [42], indicating anti-adipogenic effects. In vitro studies have reported a reduction in lipid synthesis genes (FASN and ACC) [30,34,42,43,49], and others report a reduction in lipid accumulation and glycerol release [28,30,32,33,34,36,41,42,43,46,49]. These findings collectively suggest a diminished ability for lipid synthesis and storage in adipocytes. Moreover, many in vitro studies reported the browning of white adipocytes and enhanced thermogenic gene levels [32,39,41,47], accompanied by elevated oxygen consumption [32], suggesting increased cellular respiration and thermogenic function. Similarly, in vivo studies reported reduced fat mass, adipocyte size, adipocyte number, and lipid content in WAT [32,33,53,54,57,58,59,61,62,64,68,70]. Others reported a reduction in plasma pro-inflammatory markers in adipocytes [29,37,53,58,66], and the induction of thermogenesis genes and increased browning [32,39,54,57,61,62,65,70] in animals in vivo.

**Figure 4:**
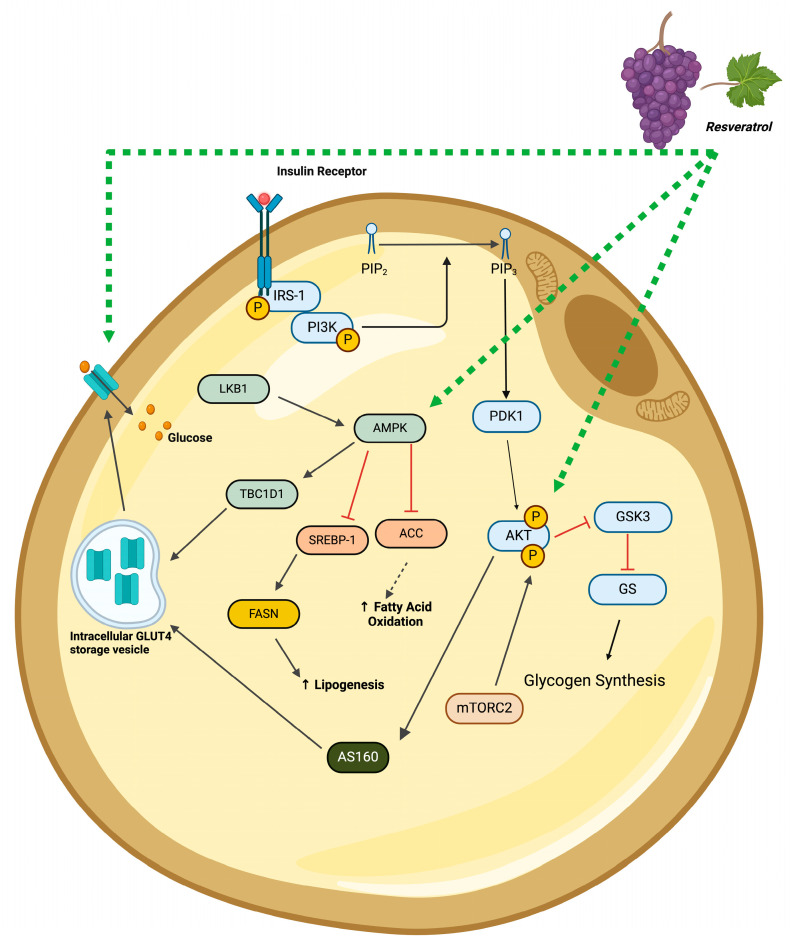
Summary of the insulin-like effects of resveratrol in adipocytes. The figure, created using www.BioRender.com, is based on the data of the studies [29,32,33,34,40,48,49,57,58,60,70,71,73]. ↑: increased, p: phosphorylated, black arrow: established pathway, green line/arrow: stimulation, red line/arrow: inhibition.

A study demonstrated an insulin-sensitizing effect of RSV, indicated by the attenuation of fatty acid-induced insulin resistance [48], and others report a reduction in the pro-inflammatory responses [29,35,48,49,52]. Three in vitro studies reported that RSV increased the adipocyte plasma membrane levels of GLUT4 [29,49,52] and glucose uptake [40,49] (Figure 4). In another study [34], researchers found significantly decreased insulin-induced phosphorylation/activation of AKT, with resveratrol suggesting the inhibition of insulin signaling. it should be noticed that limited studies have examined the effect of resveratrol on insulin signaling and its response. Thus, this controversial finding [34] should be further explored in future studies. Despite not having a clear understanding of the effects of resveratrol on insulin signaling, the evidence from in vivo studies suggest an improved insulin sensitivity and glucose tolerance [37,39,54,58,59,67,68,70] following resveratrol supplementation, associated with improved IRS1-PI3K-AKT [29,58] signaling in adipocytes.

A few in vitro studies indicate that RSV increases the phosphorylation/activation of AMPK [32,46] and SIRT-1 [31,36,39] levels, while the knockdown of AMPK and SIRT-1 abolishes the effects of RSV [36,39]. This in vitro evidence suggests a role for AMPK in mediating the effects of RSV and aligns with in vivo studies demonstrating the phosphorylation/activation of AMPK in the adipose tissue of obese insulin-resistant/diabetic animals supplemented with RSV [32,46,57,60]. Utilizing AMPK-specific in vitro and in vivo knockdown/out approaches is necessary to elucidate the role of AMPK. The same approach can be applied to elucidate the role of SIRT-1 and other signaling molecules involved in the mechanism of action of RSV.

Resveratrol has been thought to have low absorption and undergo rapid metabolism, resulting in low bioavailability, a potential limitation for treatment applications [75,76,77]. Despite the low bioavailability of resveratrol, early studies in humans indicate that significant plasma concentrations of unmetabolized resveratrol (1.8 to 2 µM) could be reached [75,78,79]. As aforementioned, in vitro studies using micromolar concentrations of RSV have shown significant anti-obesity and anti-diabetic effects on adipocytes.

The limited clinical studies, summarized in this review, provide evidence that RSV administration may improve insulin sensitivity [73,74], enhance thermogenesis [39], and reduce inflammation in obese or T2DM individuals. However, additional research is required to fully understand the effects of RSV, including its potential toxicity in humans, and to elucidate the cellular mechanisms involved.

Overall, the available in vitro, animal, and clinical human studies demonstrate the anti-obesity and anti-diabetic properties of RSV. However, to better comprehend its efficacy and safety, more comprehensive animal and clinical studies are needed to explore the therapeutic potential of RSV in the context of obesity and T2DM.

## Figures and Tables

**Table 1:** Summary of the effects of resveratrol and metabolites on adipocytes in cell culture (in vitro).

Study	Cell Line	Treatment	Effects
Mouse-derived Adipocytes			
[28] (2012)	3T3-L1 Adipocytes	RSV 10, 20, and 40 µM (4 Days During Differentiation)	↓ Proliferation ↓ Lipid Accumulation and Triglyceride Content ↓ C/EBPβ, CEBPα, FABP4, MMP-2, MMP-9, and PPAR*γ*
[29] (2013)	3T3-L1 Adipocytes	Monkey Serum HFD-RSV (24 h)	↓ NFκB protein, and IL-6 mRNA levels ↑ SIRT-1 ↑ GLUT-4 on Cell Surface
[30] (2013)	3T3-L1 Adipocytes	RSV 3.1, 6.2, and 9.3 µM During Differentiation	→ Cytotoxicity ↓ Lipid Accumulation ↓ FASN
[31] (2015)	Primary Mouse eWAT Adipocytes	RSV (10 µM, 24 h) PA (100 µM, 24 h)	↓ HIF-1α Accumulation ↑ SIRT-1
[32] (2015)	Primary Mouse SVC Adipocytes from iBAT	RSV 20 and 40 µM	↓ Lipid Accumulation ↓ PPAR*γ* and aP2 Protein Levels
		RSV 10 µM	↑ p-AMPK ↑ PRDM16, UCP1, PGC1α, Cyto C, and PDH Protein Levels ↑ Oxygen Consumption
[33] (2016)	3T3-L1 Pre- and mature Adipocytes	RSV 0.03 to 10 µM 8 days	→ Cytotoxicity ↓ Lipid Accumulation ↓ Adipocyte Maturity and Differentiation ↓ PPAR*γ* and Perilipin Protein ↓ Glycerol Release (Lipolysis)
[34] (2016)	3T3-L1 Adipocytes	RSV 25, 50, 100, and 200 µM 12 days (differentiation period)	↓ Lipid Accumulation and Droplet Formation ↓ Citrate Synthase Activity (Mitochondrial Mass) ↓ ATAD3, VDAC, ATP5a, UQCR, SDHB, and MTCO1 Protein ↓ p-Akt and 4E-BP1 Protein ↑ p-ACC Protein
[35] (2016)	3T3-L1 Adipocytes	RSV 25 µM 24 h	↑ Mitochondrial ETC-Associated Proteins (Cyto C and NADH Dehydrogenase) ↓ Lipid Metabolism-Related Proteins (SCD1, EBP, and DGAT1) Attenuated LPS-Induced increase in protein glycoprotein Attenuated LPS-Induced Reduction in Adiponectin Protein Attenuated LPS-Induced Pro-inflammatory (STAT1 and IFIT1) Proteins
[36] (2017)	3T3-L1 Adipocytes	RSV 25 or 50 µM 3 days	↓ Triglyceride Accumulation ↑ SIRT-1, PPAR*γ*, CPT1a, and PGC-1α mRNA and Protein Levels Attenuation of Effects in SIRT-1 KO Model
[37] (2017)	3T3-L1 Adipocytes	RSV 200 µM 0.6 mM PA	↓ ATF6, PERK, IRE1α, GRP78, and CHOP mRNA ↓ IRE1, CHOP, and GRP78 Protein Levels ↑ Adiponectin, SIRT-1, and PPARα ↓ PA-induced Leptin, Resistin, TNF-α, IL-1β, PPAR*γ*, and SREBP1c mRNA
[38] (2019)	Primary Mouse SVC Adipocytes from iBAT	RSV Maternal	No Change in UCP1, CPT1B, PPARGC1α, PPARGC1B, PPARA, PRDM16 mRNA levels
	Primary Mouse SVC Adipocytes from iWAT		↑ PPARGC1α in SVC iBAT No change in UCP1, CPT1B, PPARGC1B, PPARA, PRDM16 mRNA levels
[39] (2019)	Primary Mouse Adipocytes	RSV 50 µM 12 h	↑ UCP1, PGC-1α, FNDC5, and SIRT-1 mRNA in Brown Adipocytes ↑ UCP1, FNDC5, SIRT-1 mRNA in sWAT ↑ SIR-1 in vWAT SIRT-1 dependency
[40] (2020)	3T3-L1 Adipocytes	RSV 20 µM 12 h PA 0.75 mM 24 h	↓ Lipid Accumulation and TG Content ↑ Glucose Uptake ↓ ROS
[41] (2020)	3T3-L1 Adipocytes	RSV 10, 20, and 40 µM	↓ Lipid Accumulation ↑ UCP1, PPAR*γ*, and PGC-1α Protein Levels ↑ p-mTOR and p-p70S6K Protein Levels
[42] (2023)	3T3-L1 Adipocytes	RSV 1, 2, 5, and 10 µM	↓ Proliferation, Lipid Accumulation TG Content ↓ PPAR*γ*, C/EBP, FABP4, FASN mRNA ↓ p-AMPK protein
[43] (2012)	3T3-L1 Adipocytes	RSV 25 µM	↓ Lipid Accumulation ↓ Adipocyte Maturation ↓ C/EBPβ (In Pre-adipocyte) ↑ ATGL, CPT-1, SIRT-1, PGC1-α (In Mature Adipocyte)
		R3G	↓ Lipid Accumulation (In Mature Adipocyte) ↓ C/EBPβ (In Pre-Adipocyte) ↓ FASN (In Mature Adipocyte)
		R4G	↓ Lipid Accumulation ↓ C/EBPβ (In Pre-adipocyte) ↑ HSL (In Mature Adipocyte)
		R3S	↓ Lipid accumulation (In Pre-adipocyte) ↓ C/EBPβ, CEBPα, PPAR*γ*, and LPL (In Pre-adipocyte)
[44] (2013)	3T3-L1 Adipocytes	RSV 10 and 25 µM	↑ Adiponectin mRNA (10 and 25 µM) ↓ Leptin mRNA and Secretion (10 and 25 µM) ↑ Visfatin mRNA (25 µM) ↑ Apelin mRNA (25 µM)
		R3G 10 and 25 µM	↑ Adiponectin mRNA (25 µM) ↑ Leptin mRNA (10 µM), ↓ Leptin secretion (10 µM) ↑ Visfatin mRNA (10 and 25 µM) ↑ Apelin mRNA (10 and 25 µM)
		R4G 10 and 25 µM	↑ Adiponectin mRNA (25 µM) ↓ Leptin mRNA and Aecretion (10 µM) ↑ Visfatin mRNA (10 and 25 µM) ↑ Apelin mRNA (10 and 25 µM)
		R3S 10 and 25 µM	↑ Adiponectin mRNA (25 µM) ↓ Leptin mRNA and secretion (10 µM) ↑ Visfatin mRNA (10 µM) ↓ Apelin mRNA (25 µM), ↑ Apelin mRNA (10 µM)
[45] (2017)	3T3-L1 Adipocytes	RSV 25 µM	↑ miR-155 ↓ CREB1, KLF5 mRNA ↓ CEBPβ Protein and mRNA
		R3G 25 µM	↑ miR-155
		R4G 25 µM	↑ miR-155 ↓ CREB1 and KLF5 mRNA
		R3S 25 µM	↓ SREBF1 mRNA ↓ IXRα mRNA No Change: miR-155, miR-27b, miR-27a, miR-130b, miR-31, miR-326, miR-144, miR-205, and miR-244
[46] (2023)	3T3-L1 Adipocytes	RSV 40 µM DR2 20/40/80 µM	DR2 Cytotoxicity ≤ 502.5 µM|RSV Cytotoxicity ≤ 162.6 µM ↓ Lipid Accumulation and Adipocyte Maturation ↓ PPAR*γ*, C/EBP1α, and FASN ↓ p-p38 and SIRT-1 ↑ p-AMPK
Rat-derived Adipocytes			
[47] (2016)	iBAT brown preadipocytes from Male Sprague Dawley rats	RSV 10, 50, and 100 µM During Differentiation	↑ UCP-1, p-AMPK, and ER-α Protein Content
Human-derived Adipocytes			
[48] (2009)	Human SVC Adipocytes From Abdominal WAT	RSV 50 µM (12 h) CLA 10 and 12 µM (12 h)	→ Cytotoxicity ≥ 99 µM ↑ Glucose Uptake ↓ Lipid Accumulation and Free Fatty Acid Uptake ↓ p-JNK, p-ERK, ATF3, and SOCS-3, ↓ p-PLA2, COX-2, and PGF2α ↑ PPAR*γ*, SIRT-1, Adiponectin, and SIRT-1 ↓ IL-6, IL-8, IL-1b, IL-6, IL-8, and IL-1b ↓ ROS
[49] (2010)	SGBS Human Premature Adipocytes	RSV 50 and 100 µM	→ Cytotoxicity ≥ 99 µM ↓ Thymidine Incorporation (Proliferation), and Survival
	SGBS Human Mature Adipocytes	RSV 50 µM	↓ Lipid Accumulation and Glucose to Lipid Incorporation ↑ Glucose Uptake ↓ GLUT4, FASN, PPAR*γ*, and ACC ↓ IL-6 and IL-8 mRNA and Secretion ↑ SIRT-1 and Abolished Effects in SIRT-1 KO Model
[50] (2013)	SGBS Human Adipocytes	RSV 100 µM MCM 48 h	↓ PAI-1 mRNA and Protein Levels ↓ PAI-1 independent of AMPK, and PI3K
[51] (2015)	SGBS Human Adipocytes and THP-1 Monocytes	RSV 0, 10, 30, 100 µM MCM 0, 5, 10, 20% 48 h	↓ IL-6, IL-8, MCP-1 PI3K Inhibitor Prevented RSV-Induced ↓ MCP-1
[52] (2017)	Human Placenta, Adipose and Muscle	200 µM RSV 10 ng/mL TNF-α, 10 μg/mL LPS or 50 μg/mL 20 h	↓ IL-1α, IL-1β, IL-6, IL-8, MCP-1 (induced by TNF-α, IL-6, IL-1β) ↑ Insulin-Stimulated Glucose Uptake ↑ p-IRS-1 and GLUT-4

Table legend: ↑: Increased, ↓: reduced, →: unchanged, p: phosphorylated, RSV: resveratrol, eWAT: epididymal white adipose tissue, iWAT: inguinal white adipose tissue, PA: palmitic acid, CLA: conjugated linoleic acid, SVC: stromal vascular cells, SGBS: Simpson–Golabi–Behmel syndrome cells, THP-1: human Tamm–Horsfall protein THP-1 macrophages, DR2: dihydro-resveratrol, R3G: trans-resveratrol-3-O-glucuronide, R4G: trans-resveratrol-4′-O-glucuronide, R3S: trans-resveratrol-3-O-sulfate, and MCM: macrophage-conditioned media.

**Table 2:** Summary of the effects of resveratrol and metabolites on adipose tissue (in vivo).

Study	Cell Line	Treatment	Effects
Mouse Adipose Tissue			
[53] (2011)	C57BL/6J Male Mice	RSV 0.4% (ad libitum) 10 weeks HFD	↓ Total body weight and visceral fat ↓ Plasma triglyceride, glucose, TNFα, and MCP1 levels ↓ GalR1/2, PKCδ, Cyclin-D, E2F1, p-ERK ↓ PPARγ2, C/EBPα, SREBP-1c, FASN, LPL, aP2, and leptin ↓ Pro-inflammatory cytokines (TNFa, IFNa, IFNb, and IL-6) ↓ Pro-inflammatory signaling (TLR2/4, MyD88, Tirap, TRIF, TRAF6, IRF5, p-IRF3, and NF-kB)
[54] (2014)	Male Swiss Mice	RSV 4 g/kg 8 weeks	↓ eWAT and rWAT → Lean mass, food intake, iBAT mass, and total mass Improved serum lipid profile (↓Total-C, and → HDL-C) ↓ Plasma glucose levels ↑ Oxygen consumption ↑ UCP1, SIRT-1, PTEN, and BMP-7 mRNA in BAT
[55] (2014)	Homozygous ApoE-Deficient Mice	RSV 0.02% (*w*/*w*)	↓ body weight gain ↓ Plasma total cholesterol and LDL-cholesterol, triglycerides, and ApoB, ApoB/ApoA-I ratio ↑ HDL-C
[32] (2015)	CD1 Mice	0.1% (*w*/*w*) RSV orally 4 weeks HFD (45% kcal fat)	↓ Body mass, WAT index (mass/body weight) ↑ UCP1, PRDM16, Cyto C, and p-AMPK Protein and mRNA in iWAT ↓ Adipocyte size in iWAT ↑ O_2_ consumption and ↓ RER
[33] (2016)	C57BL/6C Male Mice	RSV 1, 10, and 30 mg/kg 10 weeks	↓ Total body weight gain ↓ Epididymal and subcutaneous adipose tissue gain ↓ Hepatic mass (at 30 mg/kg dosage) ↓ Adipocyte size, ↑ number of adipocytes in subcutaneous and epididymal adipose tissue
[31] (2016)	ICR Male Mice	RSV 50 mg/kg	↓ Hypoxia adducts ↓ HIF-1α mRNA and protein ↓ IRE1α, EIF2α mRNA ↓ Col3α/6α, elastin, LOX mRNA ↓ TNFα, MCP-1, IL-6 F4/80 mRNA ↓ p-IREα and eIF2α protein
[56] (2017)	CD1 Female Mice	RSV 0.1% (*w*/*w*) HFD (45% kcal fat)	↓ Average daily weight gain Daily food intake and BAT mass (No Change) ↑ Metabolic inefficiency ↑ Brown adipocytes (number of nuclei) ↑ AMPK, UCP1, PRDM16, and Cyto C protein
[57] (2017)	Maternal C57BL/6J Female Mice	0.2% (*w*/*w*) RSV Orally HFD (45% kcal fat)	↓ Body mass, iWAT, and eWAT ↓ Serum triglyceride and insulin
	Offspring C57BL/6J Male Mice	RSV from lactation RSV 0.2% (*w*/*w*) (orally) for 11 weeks post-weaning	↓ Body mass, iWAT, and eWAT ↓ Adipocyte (white) size ↑ UCP1, PRDM16, PGC-1α, Elov13, and CD137 mRNA ↑ UCP1, PRDM16, Cyto C, SIRT-1, and p-AMPK protein ↑ O_2_ consumption, heat production
[58] (2018)	C57BL/6 Male Mice	RSV 200 or 400 mg/kg/day	↓ sAT and vAT mass (mass/body weight) ↑ Insulin sensitivity HOMA and glucose tolerance ↓ Plasma TC, TG, and LDL-C, ↑ plasma HDL-C ↑ GLUT4, IRS-1, and p-Akt mRNA and protein (sAT) ↓ Serum MCP-1, TNF-α, and IL-6 ↓ F4/80 protein content and mRNA ↓ IL-6 (sAT)
[59] (2018)	C57BL/6J Male Mice STZ and NA T2D Mice	RSV 0.4% HFD (60% kcal fat)	↓ Body weight gain, visceral and iWAT ↑ Insulin and glucose sensitivity, OGTT and IPITT ↓ Adipocyte size, ↑ number of adipocytes ↓ Alpha diversity, *Bacteroidetes* and *Proteobacteria* phyla ↑ *Firmicutes* phylum
		RSV (FMT) HFD (60% kcal fat)	↓ Body weight gain, iWAT ↑ Glucose tolerance (reduced OGTT AUC) ↓ Alpha diversity, ↑ SIRT-1 mRNAin vWAT and iWAT
[37] (2019)	C57BL/6J Mice	RSV (400 mg/kg/d) HFSD (20% lard and 20% sucrose)	↓ Body mass, SAT, and VAT ↑ Insulin sensitivity and glucose tolerance ↓ Serum LDL-C, total-cholesterol, FFA, and PA levels ↓ Serum IL-6, Adiponectin, TNF-α, and MCP-1 levels ↑ PPARα and SIRT-1 mRNA levels ↓ Leptin, GRP78, ATF6, PERK, IRE1α, and CHOP mRNA levels in sAT and vAT ↓ Adipocyte size
[60] (2019)	C57/Bl6 Mice	RSV 2.3 μg/kg/day 2 days intraperitoneal injection	↓ Mean respiratory quotient (VCO_2_/VO_2_) ↑ AMPK and ACC1 mRNA in WAT ↑ CACT protein in WAT ↓ Malonyl protein in WAT
[39] (2019)	FVB/N Male Mice	RSV (400 mg/kg) orally HFD (60% kcal fat) 8 weeks	↓ vWAT and sWAT weight ↑ BAT mass ↑ Glucose tolerance, insulin sensitivity ↓ Total cholesterol, and triglyceride plasma levels HDL-cholesterol (no change) ↑ FNDC5, UCP1, and PRDM16, mRNA in sWAT, and vWAT ↑ SIRT-1, UCP1, PGC-1α, and PRDM16 mRNA in BAT
[61] (2020)	db/db (Diabetic) Mice	RSV 0.4% (in the diet) 10 weeks	↑ Glucose tolerance ↓ iWAT and eWAT mass and ↑ BAT mass ↑ UCP1 mRNA and protein in iWAT and BAT ↑ Cidea, Ppara, Pparg, and PRDM16 mRNA in iWAT ↑ Cidea, PRDM16, Ppargc1a, and Dio2 mRNA in BAT altered plasma gut microbiota ↑ Plasma and feces level of Lithocholic Acid ↓ *Firmicutes*/*Bacteroidetes* (F/B) ratio
		RSV 0.4% (in the diet) 10 weeks + antibiotics (Atx)	Abolished RSV-induced effects → Thermogenic genes in iWAT and BAT → Glucose tolerance
		Transplantation (FMT) of RSV-treated mouse feces	↑ Glucose tolerance ↓ iWAT and eWAT mass and ↑ BAT mass ↑ UCP1 mRNA and protein in iWAT and BAT ↑ Cidea, Ppara, Pparg, and PRDM16 mRNA in iWAT ↑ Cidea, PRDM16, Ppargc1a, and Dio2 mRNA in BAT
[40] (2020)	C57BL/6J Mice	RSV (15 mg/kg) IP injection for 10 weeks HFD (60% kcal fat)	↓ Weight gain ↓ iWAT mass and adipocyte size ↓ FFA and glycerol levels in iWAT
[62] (2020)	C57BL/6J Mice	RSV 100 mg/kg/day oral gavage 16 Weeks	Altered microbiome (↑ *Bacteroidetes*, and ↓ *Firmicutes*)
		RSV transplantation (FMT)	↓ Daily weight gain, WAT mass, ↑ BAT mass ↓ White adipocyte size ↑ CPT-1α, PDK4, and PPARa in iBAT ↑ UCP1, PRDM16, PGC-1a, and SIRT-1 in iBAT ↓ SREBP-1c, FASN, and SCD1 in iBAT
[46] (2023)	C57BL/6J Mice	DR2 40 and 80 mg/kg 3 weeks HFD 60% of kcal 9 weeks	↓ Daily weight gain in HFD No changes to food consumption ↓ MCP1 mRNA in iWAT ↑ AMPK Protein in iWAT
Rat Adipose Tissue			
[63] (2009)	Sprague Dawley Male Rats	RSV 30, and 60 mg/kg/day 6 weeks HCD	↓ eWAT, perirenal, mesenteric, and subcutaneous adipose tissue mass → Gastrocnemius muscle or liver mass → Serum lipid profile
[64] (2011)	Sprague Dawley rats	RSV (30 mg/kg) HFD (20% sucrose, 22.5% fat)	No effect on body mass or weight gain ↓ Perirenal, subcutaneous, and eWAT mass ↓ G6PDH, FASN, ACC, and HSL mRNA Levels
[65] (2013)	Sprague Dawley Male Rats	RSV 30 mg/kg/day (in the diet) 6 weeks HFSD	↑ Mitochondrial biogenesis (TFAM, COX2, PPARα/β, and PGC-1α) mRNA in iBAT ↑ SIRT-1 mRNA in iBAT ↑ UCP1 protein levels in iBAT
[66] (2016)	Wistar Male Rats	RSV 500 mg/kg (added to chow)	↓ Body weight gain ↓ Omental fat mass ↓ Plasma insulin, TG levels ↓ Adipose insulin levels ↓ Adipose MDA, IL-6, IL-10, IL-18 ↓ Adipose PI3K, iNOS mRNA ↓ Nrf2
	Female Wistar Rats	RSV 500 mg/kg (added to chow)	↓ Omental fat mass ↓ Plasma insulin, TG levels ↓ Adipose insulin levels ↓ Adipose TNF-α, ALT, AST ↓ Adipose MDA, IL-6, IL-10, IL-18 ↓ Adipose PI3K, iNOS mRNA ↓ Adipose Akt, eNOS, PPAR*γ* mRNA
[67] (2018)	Wistar Male Rats STZ and NA	RSV (5 and 10 mg/kg) 30 days	↓ Blood glucose, plasma insulin levels, and HOMA ↑ SOD activity ↓ FOXO1 and FOXO3 mRNA levels
[68] (2018)	Wistar Male Rats	RSV 200 mg/kg (orally) 9 weeks	↓ Body weight, fat accumulation ↓ Total levels of lipids in liver, muscle, and eWAT ↓ Serum leptin, glucose, insulin, and triglycerides ↓ Activity of ACC, SCD1, FAS, and leptin mRNA levels ↑ SIRT-1 levels in liver and muscle, but not eWAT ↑ SIRT protein levels in isolated tissue samples
[69] (2019)	Wistar Male Rats	RSV 30 mg/kg/day 6 weeks HFSD	↓ WAT mass gain ↑ NOV/CCN3 mRNA levels in eWAT
[70] (2021)	Wistar Male Rats	RSV ligand-coated 200 mg/kg encapsulated nanoparticles (biweekly)	↓ Body weight, total fat mass, gWAT and iWAT ↑ Insulin sensitivity ↓ Fasting plasma insulin and glucose concentrations ↓ Adipocyte size in iWAT, ↑ UCP1 protein in iWAT ↓ Plasma leptin concentrations, total cholesterol, and LDL cholesterol ↑ Detection of phase II conjugates and glucuronides metabolites
Monkey Adipose Tissue			
[29] (2013)	Rhesus Male Monkeys	RSV 40 mg twice daily 12 months 240 mg twice daily 12 months HFSD (27% sucrose, 42.3% fat) 24 months	↓ Adipocyte size, ↑ Adipocyte number in vWAT ↓ NF-kB, IL-6 and IL-1β mRNA in vfat ↑ IRS1, p-Akt, Glut-4, and SIRT-1 protein ↓ Serum LDL-Cholesterol

Table legend: ↑: increased, ↓: reduced, →: unchanged, and p: phosphorylated, RSV: resveratrol, w/w: weight/weight, HFD: high-fat diet, ad libitum: at the animals’ pleasure, STZ: streptozotocin, NA: nicotinamide, eWAT: epididymal white adipose tissue, iWAT: inguinal white adipose tissue, sWAT: subcutaneous white adipose tissue, vWAT: visceral white adipose tissue, iBAT: interscapular brown adipose tissue, ApoE: apolipoprotein E, HFSD: high-fat–sugar diet, FMT: fecal microbiota transplantation, Atx: antibiotics, IP injection: intraperitoneal injection, and DR2: dihydro-resveratrol.

**Table 3:** Summary of the effects of resveratrol on adipose tissue of humans (clinical studies).

Study	Subjects	Treatment	Effects
[72] (2012)	Humans (n = 45) Female Postmenopausal Non-obese Normal Glucose Tolerance	RSV 75 mg/kg (Orally) 12 Weeks	→ Body composition, metabolic and inflammatory markers → SIRT-1, NAMPT, PPARGC1A, and UCP3 mRNA levels in muscle → p-AMPK protein levels
[71] (2018)	Humans (n = 13) Male With T2D	RSV 150 mg/kg (Orally) 30 Days	↑ Plasma RSV ~300 ng/mL ↑ Plasma dihydro-resveratrol ~600 ng/mL → Insulin sensitivity ↑ Mitochondrial function in muscle → Ectopic fat accumulation, and cardiac function → Brown adipose tissue activation and function
[73] (2018)	Human (n = 28) Obese Males Aged 30–70 Years Old (BMI ≥ 30 kg/m^2^)	Trans-RSV 1 g (Orally, Twice Daily) 30 Days	↓ Glucose level in GTT (Cauc. pts.) ↑ Insulin sensitivity (Cauc. pts.) ↑ Relative abundance of Akkermansia muciniphila, Barnesiella, and Odoribacter (Cauc. pts.)
[39] (2019)	Human (n = 20) Males Aged 30–55 Years Old (BMI ≥ 30 kg/m^2^)	Trans-RSV 500 mg (Orally) 4-Weeks	↑ UCP1, PRDM16, and SIRT-1 mRNA in subcutaneous adipose tissue
[74] (2020)	Human Mixed-Gender Study Aged 30–60 Years Old (BMI ≥ 30 kg/m^2^)	RSV 250 mg/day (Orally) 3 Months	↓ Body weight, BMI, waist circumference, fat mass ↓ Systolic and diastolic blood pressure ↓ LDL cholesterol, triglycerides ↑ HDL cholesterol ↓ Fasting glucose level ↑ Insulin sensitivity (HOMA-IR) ↓ Inflammatory markers (TNF-α, CRP, IL-6) ↓ Oxidative stress markers (MDA) ↑ Anti-oxidant activity (SOD, GPx)

Table legend: ↑: increased, ↓: reduced, →: unchanged, p: phosphorylated, T2D: type 2 diabetes, BMI: body mass index, and n: sample size.
